# Role of Food Hydrocolloids as Antioxidants along with Modern Processing Techniques on the Surimi Protein Gel Textural Properties, Developments, Limitation and Future Perspectives

**DOI:** 10.3390/antiox11030486

**Published:** 2022-02-28

**Authors:** Noman Walayat, Jianhua Liu, Asad Nawaz, Rana Muhammad Aadil, María López-Pedrouso, José M. Lorenzo

**Affiliations:** 1 College of Food Science and Technology, Zhejiang University of Technology, Hangzhou 310014, China; nomanrai66@zjut.edu.cn; 2 Shenzhen Key Laboratory of Marine Microbiome Engineering, Institute for Advanced Study, Shenzhen University, Shenzhen 518060, China; 007298@yzu.edu.cn; 3 National Institute of Food Science and Technology, University of Agricultural, Faisalabad 38000, Pakistan; muhammad.aadil@uaf.edu.pk; 4 Departamento de Zooloxía, Xenética e Antropoloxía Física, Universidade de Santiago de Compostela, 15872 Santiago de Compostela, A Coruna, Spain; 5 Centro Tecnolóxico da Carne de Galicia, Rúa Galicia No. 4, Parque Tecnolóxico de Galicia, 32900 San Cibrao das Vinas, Ourense, Spain; jmlorenzo@ceteca.net; 6 Facultade de Ciencias, Universidade de Vigo, 32004 Rua Doutor Temes Fernandez, Ourense, Spain

**Keywords:** surimi, additives, processing, textural properties

## Abstract

Texture is an important parameter in determining the quality characteristics and consumer acceptability of seafood and fish protein-based products. The addition of food-based additives as antioxidants (monosaccharides, oilgosaccharides, polysaccharides and protein hydrolysates) in surimi and other seafood products has become a promising trend at an industrial scale. Improvement in gelling, textural and structural attributes of surimi gel could be attained by inhibiting the oxidative changes, protein denaturation and aggregation with these additives along with new emerging processing techniques. Moreover, the intermolecular crosslinking of surimi gel can be improved with the addition of different food hydrocolloid-based antioxidants in combination with modern processing techniques. The high-pressure processing (HPP) technique with polysaccharides can develop surimi gel with better physicochemical, antioxidative, textural attributes and increase the gel matrix than conventional processing methods. The increase in protein oxidation, denaturation, decline in water holding capacity, gel strength and viscoelastic properties of surimi gel can be substantially improved by microwave (MW) processing. The MW, ultrasonication and ultraviolet (UV) treatments can significantly increase the textural properties (hardness, gumminess and cohesiveness) and improve the antioxidative properties of surimi gel produced by different additives. This study will review potential opportunities and primary areas of future exploration for high-quality surimi gel products. Moreover, it also focuses on the influence of different antioxidants as additives and some new production strategies, such as HPP, ultrasonication, UV and MW and ohmic processing. The effects of additives in combination with different modern processing technologies on surimi gel texture are also compared.

## 1. Introduction

The quality of protein gel used in food development depends on its functional and nutritional properties. In recent decades, there has been a growing need for protein-based products among individuals due to the increasing population [1]. Scientists are looking for food-based additives and advanced processing techniques to enhance the quality, structure, oxidation and textural attributes of surimi gel products [2,3]. With regard to food textures, it takes a profound understanding of the elements that can interface with one another and how these interactions enhance the functional and structural attributes [4,5]. The texture of meat-based products is an important sensory feature regarding consumer product assessment. Over the past few decades, there has been increasing scientific interest in the field of surimi gel characteristics, such as functional, structural and textural properties. The principal goal of studying food texture is either to enhance the texture or to develop gel products with a special texture to satisfy the needs of particular classes, such as individuals who have difficulties in eating common food items [6]. In addition, a decline in textural properties predicts the degree of oxidation in surimi and surimi-based products. Fish-based gel products are generally made from minced or cut muscle with or without added ingredients, such as snacks, sausages and kamboka gel in the seafood industry. Surimi-based gel products may have imitation appearances that indicate high value and consumer acceptability. The physical and chemical properties of surimi gel can be regulated by interactions of protein molecules and stability during processing [7].

The number of commercial gel products is growing with the use of different additives, such as monosaccharides, oligosaccharides, polysaccharides and protein-based hydrolysates. The addition of such additives has improved the gelling properties of surimi gel in different ways: (a) by preventing the protein oxidation, denaturation and aggregation; (b) by improving the intermolecular binding interactions; (c) by enhancing the amino acids cross-linking and (d) reducing the amount of free water molecules during processing and preservation [8]. Meanwhile, researchers are now focusing on the addition of novel and effective additives with modern process techniques to improve the surimi gel functional and gelling properties during processing and preservation compared to conventional methods. However, these conventional technologies are not time, cost and quality efficient to develop proper surimi gel, thus suggesting the use of modern processing techniques.

Non-conventional food technologies have been used recently to develop seafood and surimi-based products [9]. The advantages of new technologies over traditional processing techniques include low cost, time-saving, high-quality products with the least protein and lipid oxidation and full nutrient retention with marginal organoleptic changes [10,11]. The application of advanced techniques dramatically facilitates the interaction of proteins by increasing the reactive site structure and exposure level of protein molecules [12]. The development and functionality of surimi gels could be enhanced with high-pressure processing (HPP), although the transition in gelling behavior with HPP depends on its processing time, temperature system configurations, HPP-treated proteins typically form elastic and time-stable gels with more heterogeneous interconnections in the presence of food-based additives [13,14]. Apart from the HPP, the effects of microwave (MW) [15,16] and ultraviolet light (UV) [17] on surimi gelation have been widely studied. The use of these non-conventional techniques can increase the functional characteristics of surimi gels by increasing the covalent activity of protein molecules [12]. The aim of this study is to illustrate surimi gel based on different antioxidant additives and their roles in improving gel textural properties during processing and preservation. Moreover, the effects of antioxidants combined with advanced processing techniques were reviewed in detail on gel textural and protein oxidation. In addition, a comparative analysis is also highlighted between certain non-conventional and traditional processing techniques to recognize their influence on the textural attributes of surimi gel.

## 2. Fish Protein-Based Gel Products

### 2.1. Heat-Induced Gel

A number of publications on surimi thermal gelation are available. Surimi gelation is the most popular method in the seafood industry for gelling products. Surimi gelation involves myofibrillar proteins (MPs), particularly myosin. It occurs when enough salt is added to allow MPs to unfold, which causes the exposure of their reactive sites, encouraging contact, so that intermolecular bonds are formed. When such interactions are sufficient, a three-dimensional network is developed, which leads to gel formation. Various bonds, such as hydrophobic, covalent hydrogen and ionic interactions are important in the formation of dense and three-dimensional gel networks [2,18]. It is imperative to ensure that covalent and hydrophobic bindings are responsible for gel thermos stability, which is important for gel formation. If surimi is held at a low temperature (0 to 50 °C following salting), MPs can develop a more reconfigurable and softer gel network, known as suwari gel; this process is called suwari. This kind of gel is generally produced by the enzymatically catalyzed effect of transglutaminase (mTGase), which is naturally occurring in fish muscles [19]. The temperature at which proteins are unfolded and connected all depends on the species. Better results were obtained in cold-water fish at a temperature of approximately 25 °C, while suwari gels made with a water bath showed better mechanical properties when incubated at a temperature higher than 40 °C [20]. An alternative means of developing suwari is through high-pressure denaturation of protein, in which case a gel with a shinier texture is obtained. Hydrophobic interactions play a predominant role in such conditions. After heating suwari gels at 80–90 °C, stronger gels formed, while pre-incubation was performed at low temperatures [21].

A variety of ingredients and additives have been used to improve the gel texture during gelation. These ingredients can function either as protein interactors or as protein network fillers [2]. Moreover, advanced techniques (ohmic, MW and HPP) have been reported on for the heat gelation of surimi gel and preferred over the conventional techniques in literature [22,23]. The goal of such research findings was to decrease proteolytic activity and its negative impact during slow heating. Moon, et al. [24] noted that Alaska Pollock surimi gels cooked at slow ohmic heating showed increased shear stress and strain values, while Pacific surimi whiting, which has a more proteolytically active effect, showed higher levels of shear stress when heated rapidly. Two Japanese producers have commercially introduced the ohmic cooking technique for surimi gel preparation [25]. Gao, et al. [26] found that the rapid heating of low salt surimi from *Hypophthamichthys molitrix* mince during gelation prevented the protein autolysis and contributed to a superior gel strength and texture relative to traditional heat gelation. Gelation using radio frequency (RF) is a dielectric heat equivalent to processing with a MW. Helena, et al. [19,27] studied its use in gel products. These researchers demonstrated that RF heated surimi gel showed better water holding capacity (WHC) properties along with compact structure, but was considerably harder and chewier with a lower development of color than the steam cooked method. Sampels [28] reported increased water binding and gel strength of RF and MW during Atlantic salmon and Rainbow trout surimi gel. The fracture and deformation values in puncture experiments were higher when RF was being used as compared to MW when heating at varying temperatures. Furthermore, no commercial RF or MW is still available on a continuous basis.

### 2.2. Cold Gelation

In recent decades, the demand for moderately processed products has grown. In this regard, the advancement of cold gelation technology is becoming an important choice in producing surimi gel products. Products produced by this process are highly flexible and can be sold in a number of ways, including ready-to-serve fish fillets, small fillets, marinated, carpaccio-like sushiors and even smoked products [19]. There are different types of binding agents, such as alginates and mTGase available in the food industry that make surimi-based gels, possibly using cold gelation technology. In cold gelation, the aggregation of proteins is primarily due to the activity of various bindings capable of acting at low temperatures without altering the aspects of the gel formation [29]. In addition, it is also possible to produce different value-added gel products by using these bonding agents [2]. Alginate and Transglutaminase—two binding additives—improve the gelling and functional properties of gel products by enhancing cross-linking in amino acid side chains. However, scientific literature has recently recommended the inclusion of konjac glucomannan (KGM) into surimi to allow the gel to be made from non-functional raw material.

### 2.3. Salt-Based Surimi Gel

The desired surimi gel can be acquired by adding various salts, usually, sodium chloride (NaCl), which aid in unfolding MPs during the gelation phase [30]. Currently, salt in surimi is becoming an important concern, since decreased salt levels have reduced MP solubilization and thereby contributed to weak surimi gel [31,32]. Different salts, such as Na^+^, Mg^2+^, K^+^ and Ca^2+^, have been stated to be alternatives to each other but often contain unacceptable flavors [33]. Li, et al. [34] stated that MPs dissolved in 2–4 g/100 g of NaCl to develop a proper gel network. In comparison, additional additives in conjunction with HPP and reduced addition of sodium chloride would also contribute to physicochemical properties close to normal surimi gel as after the addition of normal salt amount.

## 3. Role of Antioxidants in Improved Surimi Gel Textural Properties

### 3.1. Carbohydrate-Based Gel

There are different food-based antioxidants, such as carbohydrates, proteins and peptides, that play a significant part in surimi gel products as effective gelling agents during preservation and processing. In addition, these have been proposed with improved antioxidative, textural, gelling and functional properties in heat-induced gel [2]. Moreover, these develop the proper gel matrix, improve the viscosity of protein solution and enhance textural and structural attributes. The role of different additives in the enhanced surimi gel properties is shown in Table 1.

#### 3.1.1. Saccharide-Based Gel

Walayat, Xiong, Xiong, Moreno, Nawaz, Niaz and Randhawa [2] identified the effect of saccharides as cryoprotectants on protein-based gel in three ways. Firstly, monosaccharides, oligosaccharides and polysaccharides have strong buffering properties that assist with thick filament depolymerization. Secondly, they bond with myosin molecules, whilst strengthening actomyosin disassociation by contrast to ATP and binding to the myosin head. Thirdly, myosin tail-linked saccharide molecules strengthen the disassociation of myosin filaments. In the whole mechanism, saccharides also work as effective antioxidants, which prevent protein denaturation and aggregation during preservation and processing [35].

**Figure 1:**
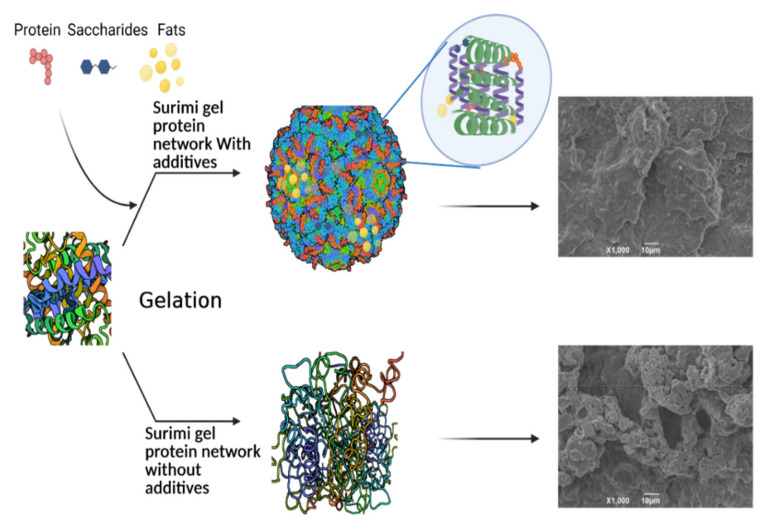
Surimi gelling properties with and without incorporation of additives. Microstructure figures reprinted from Walayat, Xiong, Xiong, Moreno, Li, Nawaz, Zhang, Wang and Niaz [8].

An illustration of surimi gelling properties with and without the incorporation of additives is shown in Figure 1. Sucrose is an essential cryoprotective and antioxidant agent typically used in surimi during frozen preservation. It is usually added at 4% in combination with different additives, such as sorbitol and phosphates during the frozen storage of seafood, fish-based hydrolysates and mince to prevent the denaturation and aggregation of MPs [36]. Commercial mixtures of sucrose with polyphosphates have been proven to reduce protein oxidation of surimi proteins and improve the gelling abilities by forming proper cross-linking between the amino acids [37,38]. Moreover, the addition of sucrose with other saccharides enhanced the water binding and textural properties of surimi gel. The sorbitol and hexametaphosphate blend also enhanced the Alaska Pollock texture and water retention properties [39]. Polydextrose is a dry, odorless, less sweetened and amorphous powder. Polydextrose is an excellent stabilizer, incorporating cryoprotective and antioxidative properties when added 8% in surimi gel [40]. Moreover, litesse is an enhanced flavored polydextrose formula with additional processing to reduce polydextrose bitterness. It has also been reported that litesse successfully stabilizes the surimi gelling properties by inhibiting the oxidative changes [41]. Lactitol is a polyol-disaccharide-derived product of lactose, which is used in the surimi gel to enhance the cryoprotective and gelling properties [42]. Maltodextrin is a commonly accessible agent in various molecular weights and has been reported to have increased functional and gelling properties, as well as to reduce the oxidative changes in protein-based seafood products [43]. Moreover, sodium lactate is also an FDA-approved protein additive used in surimi and other seafood products during frozen storage. Sodium lactate prevented the drip loss and decline in textural properties of surimi heat-induced gel, which was further proved by the enhanced storage and loss modulus [44]. Trehalose is well-known as non-reducing sugar, which was reported by other studies [2,45] as a fish-based protein additive, which efficiently improved the storage stability of gel products by inhibiting protein oxidative, denaturation, secondary and tertiary structural changes. Various kinds of phosphates have been reported in heat-induced gel formation, such as sodium triphosphate, trisodium phosphate, sodium pyrophosphate and tetrasodium pyrophosphate. These saccharides are typically added in low concentrations (0.5%) to surimi and seafood gel products in combination with different hydrocolloids and protein-based additives for the enhanced and stable gel network during preservation and processing [46]. Meanwhile, higher incorporation could also affect the structural and gelling attributes by chelating ions important for the mTGase enzymatic activity [47].

#### 3.1.2. Other Polysaccharide-Based Surimi Gel

Carbohydrates improve the surimi gel characteristics by interfering with protein interaction sites. The incorporation of carbohydrates into fish protein is encouraging, leading to low protein salt solubilization and a drop in the functional characteristics of the gel. During high-temperature processes, starch increases gel strength, whiteness and thermal stability [48,49]. Mi, et al. [50] noted that the inclusion of surimi gel with pregelatinized starch increased the gel rigidity. Moreover, rice starch improved the textural and WHC properties of surimi gel during the gelation process [18]. Gum also provides better mechanical and textural features as well. Some gums have strong fish protein compatibility, thereby improving the heat-induced gel textural properties [51]. Liu, et al. [52] have reported that KGM is effective against oxidation/denaturation/aggregation of MP proteins during frozen storage. KGM in surimi regulated the WHC and gel strength. Ye, et al. [53] stated that surimi gelling characteristics were enhanced by adding carrageen and konjac during frozen storage by enhancing the antioxidative abilities and reducing the oxidation-based protein denaturation. Moreover, pectin such as high-methoxyl (HM) and low-methoxyl (LM) enhanced the textural and functional attributes of surimi gel by increasing pectin polarity. Therefore, hydrogen bonds between proteins and pectin could increase the overall surimi gel characteristics. Besides that, the addition of LM pectin increased the toughness while reducing the cohesiveness of the surimi gel texture [54]. Fibers were reported as a gelling agent in the heat-induced gel of surimi. Fibers, such as red and white raisins dietary fibers (GDFs) have improved the water binding, structural and microstructural properties of horse mackerel surimi gel, generally associated with textural attributes [55]. Chicory root fibers also improved shear strain and decreased gel deformation [56]. Furthermore, wheat fiber add-on (6 g/100 g) influenced the surimi gel’s textural and structural properties. In the meantime, gel elasticity has also been improved [57]. Yin, et al. [58] reported that the addition of nano-sized okara insoluble fiber (0.8 g/100 g) enhanced the breaking force and gel integrity of silver carp gel by inhibiting the movement of free water molecules and filling the interracial spaces of the protein matrix.

### 3.2. Protein-Based Gel

Textural and structural properties during preservation and processing are adversely affected by MP denaturation. Beef plasma protein (BPP) and egg white protein (EWP) have been widely studied to inhibit the oxidative changes in surimi gel texture during preservation and processing [8,59]. These food additives are also known as proteinase inhibitors. BPP has been reported as an effective oxidative, gelling and emulsifying agent. Meanwhile, BPP contains 70% protein content with outstanding gelling properties; when cooked at 65 °C, it gives an elastic and permanent gel network [60]. Walayat, Xiong, Xiong, Moreno, Nawaz, Niaz and Randhawa [2] demonstrated that the incorporation of BPP (0.5%) in surimi inhibited the myosin degradation and enhanced the hardness and gumminess of heat-induced gel. In addition, BPP also increased the surimi gelling and textural characteristics, since BPP also comprises polypeptides that can help to form a gel. Beef plasma proteins of up to 3% reinforced lizardfish gel formation properties as compared to egg proteins [60]. EW is being used in seafood products as antioxidants, gelling, emulsifying and foaming agents. The addition of EW in surimi products is mainly based on the species and concentration of protein. Surimi added with 10% of EW exhibited an increase in gel force during frozen storage by inhibiting the oxidative process. In the meantime, 20% decreased gel resistance and a smoother gel texture in surimi were observed. In addition, EW plays a significant part in the surimi gel structure by proper cross-linking of amino acid side chains [8,59]. HenriquesHENRIQUES, et al. [61] have shown that the addition of whey protein concentrates (WPCs) to surimi improve the textural attributes of surimi gel. WPCs are widely used as water-binding agents, emulsifying, gelling and thickeners. Researchers have formally recorded a rise in the shear strain of surimi gels after incorporating WPC. As the concentration of WPC in kamaboko gel increased (0–3%), the breaking strength and deformation also increased. However, the whiteness of the gel decreased. Furthermore, the surimi gel water bonding capability was improved by the rising degree of WPC [62,63].

### 3.3. Protein Hydrolysate-Based Gel

Protein hydrolysates are effective antioxidants and are generally known as substitutes for proteins, which are usually impaired by the use of 3–20 amino acids. Several studies have been published on the role of protein hydrolysates in the improved gelling and textural properties of surimi gel by inhibiting the free radical and scavenging abilities during frozen storage [2,64]. Zhang, et al. [65] have shown that protein hydrolysates improve the gelling attributes by decreasing the water movement into protein molecules, protein oxidation, inhibiting free radicals and suggesting a more stable protein structure. The hydrolysates derived from the unicorn leather jacket skin comprised a higher concentration of hydrophilic amino acids, which may enhance the antioxidative abilities and enhance the functional properties of surimi gel by limiting the movement of water molecules in proteins, thereby reducing the crystallization process [66]. Gelatin tripeptide avoided the denaturation of myosin and ultimately reduced water loss from MPs [67]. Krill protein hydrolysates were documented with strong antioxidative abilities, which increased the functional properties of surimi gel by reducing free radicals, the formation of ice crystals and inhibiting decline in Ca^2+^ ATPase activity during frozen storage. Krill hydrolysate protein also enhanced the amount of unfrozen water in protein molecules by increasing the surface tension of protein molecules [68].

**Table 1:** Role of different additives in the enhanced surimi gel properties.

Additives	Role	Results	Reference
Sucrose and sorbitol	Saccharids, cryoprotectants and functional	Improved the structural properties, conserving three dimensional and improving the protein stability in common carp surimi	[69]
mTGase	Microbial and protein cross-linker	Increased the surimi gel strength and viscoelastic attributes by improving the intermolecular cross-linking of protein molecules	[70]
Sulfated polysaccharide (SP)	Antioxidants, nutritional and functional	The addition of SP enhanced the textural and water-holding properties of silver carp surimi gel by reducing the oxidative changes	[71]
Fucoidan polysaccharide	Antioxidants and antibacterial	Fuoxidan polysaccharide enhanced the hardness, gumminess and water-binding characteristics of surimi gel by promoting the cross-linking of protein molecules.	[72]
Kappa carrageenan (KC)	Polysaccharide, antioxidant and cryoprotectant	Addition of KC in surimi gel enhanced the gel strength and textural properties. Meanwhile, improved the viscoelastic properties.	[73]
Konjac glucomannan (KGM)	Oligosaccharide, antioxidant and functional	Incorporation of KOG increased the textural and antioxidative properties by inhibiting the movement of free water molecules.	[52]
Skipjack roe protein hydrolysate (SRPH)	Antioxidant and emulsifier	SRPH restarted the protein and lipid oxidation in sausage.	[74]
Protein hydrolysate (PH)	Antioxidants, physiological and protein functional enhancer	PH addition in silver carp surimi gel reduced the protein oxidative changes and enhanced the gel-forming abilities.	[74]

## 4. Why the Textural Properties of Surimi Gel and Oxidation Effect Are an Important Concern

Texture properties play a significant role in the quality and functionality of surimi gel. Texture analysis is vital owing to its key association with oxidative, structural, functional and microstructural attributes [75]. Currently, increasing interest has been found in research to retain the quality and functional attributes of surimi gel. Researchers are focusing on the role of different additives and modern processing techniques to improve the textural properties of surimi gel by inhibiting the oxidative, denaturation and aggregation loss of protein molecules during processing and preservation. Up until now, many studies have been published on the addition of saccharides, polysaccharides and protein hydrolysates to improve the overall quality and functional, structural and gelling characteristics of surimi gel. These characteristics depict the stability of surimi gel products. Processing and preservation are the major factors that could affect the functional and textural properties of heat-induced gel. Meanwhile, fish species, hydrocolloids and processing techniques are the key aspects that can increase the mechanical and textural properties of surimi gel products [2,76].

The reasons for the change in fish muscle texture during preservation and processing have been reviewed but several future studies are lacking. MPs and collagen polypeptides accumulate during the gelling process, thus increasing the toughness of the final gel product [77]. When proteins undergo oxidation and denaturation, it results in the toughening of gel texture, which is the most possible outcome. It collapses in the sarcoplasmic reticulum and ends up becoming a gel that binds the individual myofibrils together during the gelling process [78,79]. The oxidation of the proteins continued more steadily as the temperature kept reducing. The rate of change in protein increases as the refrigeration temperature increases. During the analysis of frozen cod fillets stored at −20 °C or below, the findings revealed that the unfolding in myosin and actomyosin occurred and reduced the mechanical and textural properties due to the enhanced oxidative changes based on protein molecule alteration [80]. Nikoo, et al. [81] analyzed that the addition of tetrapeptide in surimi proteins decreased actin and myosin during frozen storage, which is associated with oxidation and ultimately reduces textural and structural properties. The incorporation of peptides also significantly improved myosin stability by reducing carbonyls and disulfide formation. Furthermore, frozen storage also decreased the textural properties of surimi gel due to the irregular formation of ice crystals and recrystallization [37]. Besides this, processing could also result in alteration of the chemical nature of protein and release of amino acids and peptides, which further leads to the destruction of structural and functional properties [82]. For instance, carbonyl, sulfhydryl and surface hydrophobicity are among these structural changes and directly associated with the oxidation process, which generally occurs due to oxidation of myosin proteins [75]. Carbonyls are an important analysis for determining the oxidative changes that occur during seafood processing [83].

In addition, mTGase could also affect the quality of surimi protein, the gadoid fish is different because it contains the mTGase enzyme that metabolizes trimethylamine oxide into dimethylamine and formaldehyde. This enzyme is generally present in a few species other than gadoids, which means it is limited to species like red hake and cod. Formaldehyde causes cross-linking between the proteins and makes the muscles more brittle. The enzyme becomes more active when the tissues are disrupted by temperature changes [84]. Besides that, the taste of fish may also be influenced by salting and smoking. Owing to dehydration, these muscles may get firmer. Salting and smoking also significantly impact the texture of catfish. The addition of salt and lengthy smoking make the fish gel texture more brittle and hard [85]. In mackerel fillet-curing, firmness or stiffness increases in combination with the decrease in water content and the increase in the solubility of sarcoplasmic proteins during the processing [86]. It was noted that the gel prepared from the *Priacanthus tayenus* showed more gel strength (1109 g). In contrast, *Priacanthus macracanthus* was reported to have less gel strength at 811 g. Moreover, the hardness of grass carp was 621 g, which was reduced to 271 g after the change in temperatures. Meanwhile, during preservation, the mechanical attributes of tilapia were also reduced from 530 to 211 g [2].

Reduced textural properties of protein-based gels have been linked with the oxidation of denatured actomyosin, which shows no further cross-linking between the amino acid side chains and resulting product with inferior gelling properties. The oxidation and denaturation of myosin during frozen storage resulted in an inadequate gel with less elasticity and viscosity [87]. Under ice storage and inadequate processing conditions, the protein solubility and gel-forming properties of grass carp would decrease [78]. Therefore, the advanced processing techniques could be used as an alternative to conventional methods to produce gel products with better functional and textural properties.

## 5. Role of Modern Technologies in Protein Gel-Based Products

### 5.1. High-Pressure Processing

High-pressure processing (HPP) is an enticing technology for preserving food with the possibility of controlling microbe load and/or enzyme production in various products [13,88,89,90]. HPP is an adjunction approach to stimulate the protein gelation phase in a properly solubilized form. Molecular interactions by additional hydrophobic and hydrogen bonds and distributional interactions, such as disulfide bindings could stabilize the established strong protein gel network [17]. HPP could be used to boost protein functionality and easily change enzyme activity. The influence of HPP on additives and the versatility of surimi gel has been more widely studied than any other advanced developments in food processing, such as ohmic heating, MW, ultrasonication and UV light. The majority of these studies have focused on the gel development of chicken, pork plasma, surimi and turkey meat [15]. A primary consistency index for protein gel products, the WHC, is strongly influenced by the quantity of free and trapped water [91].

HPP also prevents oxidative changes in seafood and other meat products to maintain their textural and nutritional properties. Pork was stored for 8 days at 2 °C after the HPP application. At the end of the study, no significant formation of lipid oxidation was analyzed. On the other hand, HPP also prevented oxidative changes by increasing the sulfhydryl content and inhibited the formation of carbonyls, which resulted in better textural properties [5]. Morton, et al. [92] also reported that HPP significantly reduced the oxidative changes in beef, in which the oxidative changes were more stable as compared to chicken meat. Cava, et al. [93] stated that the application of HPP treatment (200 to 300 MPa) enhanced the stability of lipid and protein oxidation in dry-cured meat after storage for 90 days at 4 °C.

Moreover, depending on the operating environment, protein source, protein stability rate and gelling conditions, HPP may induce positive or negative effects on the textural properties of surimi gel [22,23]. Important improvements were recorded in gel strength and WHC using HPP on mTGase-based gel for chicken meat [13] and tilapia surimi paste [94]. HPP substantially organizes protein molecules by inhibiting the oxidative changes to increase the WHC by forming hydrophobic associations and hydrogen and disulfide bindings [95]. Tsevdou, et al. [96] reported that the HPP at 450 MPa on casein micelles in the presence of mTGase improved the textural properties of surimi gel by creating a low-porosity protein matrix, the mTGase crosslinking of small micelles reduced the syneresis rate. Moreover, the hydrolysis of covalent bonds (e.g., disulfide, hydrogen, ionic and hydrophobic) can increase the solubility of protein gels. The presence of mTGases can decrease the net protein solubility due to HPP. Tabilo-Munizaga and Barbosa-Cánovas [97] reported that the addition of potato starch and egg white protein in surimi at 400 MPa of HPP improved the whiteness and textural attributes of surimi gel to 602 (g) as compared to 483 (g) in control.

This is a reality that insoluble aggregates have been formed by having clear ties between heavy myosin protein chains [94]. The breaking force is improved substantially at the same time as HPP and EW are being added [19]. The stability of protein gels improved by HPP processing is not only due to the increasing amount of protein stretching and the number of structures, but also reduced oxidative changes, the visibility of glutamyl and lysyl residues and the formation of isopeptide bonds of enzymes [98]. Tabilo-Munizaga and Barbosa-Canovas [99] stated that the surimi gel showed better textural and WHC properties after the addition of non-ionic gum in surimi during HPP processing. Furthermore, at 200 MPa, surimi gel with kappa carrageenan demonstrated improved textural and microstructural properties [100].

### 5.2. Ultrasonication

Ultrasonication is a valuable technique and has diverse applications in food items, such as mechanical cell distortions in order to enhance the retrieval of bioactive components, micro-organism inactivation and immiscible liquid emulsification [15,101,102]. Recently, low frequency (16–100 kHz), high frequency (HIU, 10–1000 Wcm^−2^) ultrasonic techniques have been developed as a fast and secure application to alter protein structures, functional and physicochemical properties [103]. Most importantly, ultrasonication is widely encouraged by researchers to enhance food quality. Ultrasonic techniques have special characteristics that make them more effective, such as microstreaming currents, turbulence, high pressure, cavitation bubbling, leading to modification of protein structural properties [104]. Gülseren, et al. [105] reported that adding phenolic compounds to proteins and treating them with ultrasonic techniques could reduce protein cross-linking and oxidation, both of which play important roles in the gelling and textural properties of seafood proteins. He, et al. [106] reported that the application of ultrasonication combined with high salt concentration enhanced the textural properties (hardness and springiness) of silver carp surimi. Meanwhile, it prevented the change in secondary structural changes by inhibiting the unfolding of α-helix content. Pan, et al. [107] reported that the addition of phenolic compounds to MPs and treatment with ultrasonication reduced the protein oxidation (surface hydrophobicity and carbonyls) by enhancing the hydrogen and hydrophobic interactions. A big rise in the yield of soy protein isolate (SPI) catalyzed by mTGases led to an improvement in WHC and textural properties after the ultrasonication technique. Therefore, ultrasonication is also helpful in raising the SPI and wheat gluten-based hydrogels by assisting in protein structural modifications [108]. Xu, Lv, Zhao, He, Li, Yi and Li [104] reported that the ultrasound treatment with diacylglycerol (DAG) enhanced the gelling attributes of golden thread surimi. In addition, ultrasound combined with (DAG) improved the structural and microstructural properties of golden thread surimi by enhancing the intermolecular interaction, hydrogen and hydrophilic bindings. The use of short-term sonication speeds up water leakage rates because of the existence of poor structural matrix protein gels [109]. In addition, the production of low-porosity, homogenous protein structures for water absorption at higher sonic times can be related to two distinct mechanisms, including: (I) an exposition through modifications in the molecular conformation of proteins to internal active polar groups on the surface and (ii) a more fitting dispersion of these functionary groups into a sonic reaction environment [103]. It has previously been observed that high-intensity ultrasonic treatment could enhance the gel strength of SPI-set gels caused by adding glucono-β-lactone [110] and calcium sulfate [111]. Gao, et al. [112] reported that the polysaccharide-added surimi protein had higher Ca2^+^ATPase and sulfhydryl content after ultrasonication, which reduced oxidative changes in the myosin globular head by stimulating hydrogen and hydrophobic interactions. Thus, this results in a more stable gel texture and structure. Ultrasonic application appears to alter the function of the protein matrix by expanding the amount of interfacial covalent interactions. Typically, hydrophobic associations in the protein gel matrix are intensified following ultrasonication [110]. Hu, et al. [113] have demonstrated that the secondary SPI textural properties of mTGase-based gels were not altered at 20 kHz and 400 W of ultrasonic treatment. However, Gharibzahedi, Roohinejad, George, Barba, Greiner, Barbosa-Cánovas and Mallikarjunan [13] reported modifications of the secondary structure of the wheat gluten-SPI gels caused by ultrasonic technique, leading to increased β-sheets, decreased α-helices and β-turns, which indicates fewer protein oxidative, denaturation and aggregation changes. Cui, et al. [114] have identified an increase in β sheet count to boost the hydrophobic surface and viscoelastic properties of protein gels. The enhanced configurations of the β-sheets also modified the protein-protein cross-linking of hydrophobic active groups. β-sheets are more capable of hydrating water molecules compared to α-helix during ultrasonic pretreatment and it provides stronger hydrogels [113]. Ultrasonication can transform the molecular protein structure from β-turns into random coils in order to improve the cross-linkage of amino acid side chains [15]. Therefore, the larger amount of inter-molecular disulfide bonds in ultrasonic-based surimi gels can also justify the rise in gel strength. The appearance of a polymer matrix with more compact and dense aggregations of multi-molecular cross-links, hydrophobic associations and inter-molecular disulfide bindings, increases the gel strength [115]. The microstructure of heat-induced gels, however, can be dramatically altered with increasing ultrasonic time and cavitational pressure, from a spongy matrix with wider irregular pores, to a more dense and homogeneous alveolar network with fewer pores by reducing myosin oxidative changes as well as enhancing the intermolecular bonding interactions [116].

Qin et al. [108] took a particular approach to explain why the increased ultrasonic approach enhanced the wheat gluten added gel. They observed that the non-covalent interactions in the gluten structure (e.g., hydrogen bonds) were attenuated by the partial expression of protein molecules. The spatial structure of gluten reportedly promotes gel strength by creating novel and different linkages/relationships in the molecular structure, particularly covalent cross-links of inter-(μ-glutamine)-lysine. The use of Na_2_SO_3_/ultrasonic pretreatment increased the power of wheat gluten gel by up to 67% of the different pretreatments (e.g., alkaline, urea and Na_2_SO_3_) coupled with ultrasonication [117]. The use of ultrasonic treatment generally results in higher protein solubility in various solvents, particularly over long periods of time. Ultrasonication increases the sum of electrostatic bonds and other non-covalent connections compared with covalent interactions in protein gel structure. This decreases the molecular weight and increases the solubility rate by hydrolyzing disulfide bonds by adding Na_2_SO_3_ into the reaction mixture [111]. Under certain circumstances, the dispersion of protein molecules on the aqueous surface allows exposure of specific functional groups by the cavitation phenomenon during the ultrasonic application [118,119].

### 5.3. Microwave (MW)

Microwave (MW) heating has been reported as a non-conventional way of heating surimi gel, which generally focuses on the MW radiation effect on protein functional and structural attributes. Jiao, Cao, Fan, Huang, Zhao, Yan, Zhou, Zhang, Ye and Zhang [3] reported that the MW heating protected proteins from oxidation, denaturing and aggregation during processing and preservation. MW heating proved to be effective against these changes in contrast with thermal hot water treatment. Feng, et al. [120] examined the effects of both water bath and MW heating on silver carp protein, which showed that the MW heating showed better stability in Ca^2+^ATPase activity and protein solubility than the proteins heated at water bath, which indicates less myosin exposure to oxidative changes and resulting denaturation. These inhibited changes also have a potential role in enhancing the gelling and textural abilities of the final product. Moreover, MW is a type of electromagnetic radiation with a frequency between 300 Hz to 300 GHz and a wavelength of around 1 cm and 1 mm. The polarization of water in food materials with MW high-frequency radiation will unfold protein molecules, breaking down non-covalent bindings, including disulfide connectivity and hydrogen bonding [22,121]. Cao, Fan, Jiao, Huang, Zhao, Yan, Zhou, Zhang, Ye and Zhang [47] stated that MW radiation could improve the efficiency and reaction rate of enzyme systems in a certain range of radiation frequencies. Qin, Luo, Cai, Zhong, Jiang, Zhao and Zheng [108] have examined the impact of MW on the gelation process of mTGase added SPI. The increase in MW power up to 700 W decreased the solubility index, gel strength, elasticity, hardness and WHC of the proteins treated with mTGase. In contrast to untreated samples, thicker, more consistent mTGase-catalytic gels with more α-helices and β-turns and fewer portions of β-sheets were given at a constant frequency (3 GHz). The protein solubility decrease in MW treated samples was due to a reduction in the free SH content of protein gels caused by mTGase. In addition, due to the effect on hydrogen, intermolecular disulfide bonds, as well as electrostatic and hydrophobic interactions a significant number of insoluble protein aggregates can limit the solubility of proteins. Protein aggregates generally form due to oxidative changes and weaker intermolecular bonding interactions. In addition, MW heating also enhanced the stability of SH content by preventing the unfolding of amino acids caused by oxidative and aggregation changes [120]. The accumulation of these insoluble aggregates of greater particle size could decrease the potential of the water to associate with protein molecules [122].

**Figure 2:**
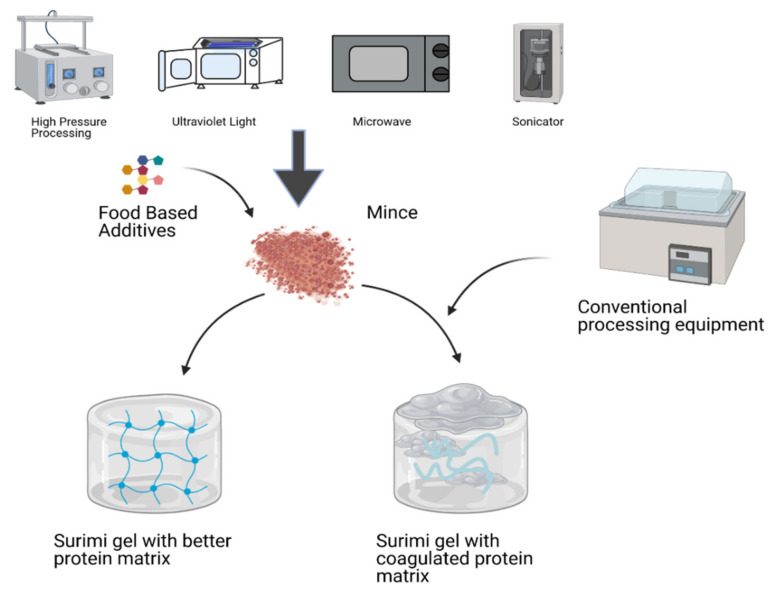
Surimi gel prepared with non-conventional and conventional techniques. Non-conventional techniques show a more stable and well-established gel network than conventional processing equipment.

The formation in MW-treated protein gels of a stable and solid well-aggregated microstructure with reduced particle size may be attributed to electrical polarization and the insoluble aggregation of proteins. The presence of this compact microstructure in surimi gels with increased gel strength and elasticity can validate improved textural properties, especially at increased MW capability [123]. Ji, et al. [124] reported that the surimi gel heated for 10 min at MW increased the breaking strength and gel firmness. As a result, it can be suggested that the surimi gelation at MW could increase the functional, microstructural and textural strength of the gel resulting from proper cross-linking of protein molecules. Thus, the resulting gel has better deformation and breaking strength as compared to the conventional gelation process. Protein behavior of surimi gel prepared with non-conventional and conventional techniques shown in Figure 2. On the other hand, MW heating not only enhances the gelling attributes but also reduces the protein matrix. The MW consistent heating system could not result in a defective gel as in the conventional heating system temperature above boiling point increases water vapor pressure, resulting in a gel with structural defects [125,126]. Besides that, the addition of konjac glucomannan (KGM) may improve the gel texture and structure by restricting the decline in protein secondary and tertiary structural properties induced during MW heating. It can also be interpreted that the addition of KGM improves the cross-linking of amino acids and reduces the MP polymerization [127]. Therefore, KGM during MW heating not only acts as a filler but is also effective in protein cross-linking and interlinking.

### 5.4. Ultraviolet

Ultraviolet (UV) light irradiation can alter the protein structure by increasing the polymerization of protein side chains. The nature of the protein and the rate of irradiation are thought to be the two most important factors influencing the formation of cross-linking aggregates or molecular structures induced by protein denaturation and oxidation during this process [128,129]. Synergistic activity between the mTGase increased the surimi gel power of minced mackerel [13]. The mechanism behind the UV irradiation is that increases the hydrophobic interactions as compared to conventional heating, which indicates less protein denaturation (oxidation and aggregation) [129]. Besides that, UV irradiation also gears up the formation of disulfide content induced during protein changes, but the formation of disulfide bonds during the UV process is less than the conventional heating methods [130]. The UV irradiation process is consistent with stability in sulfhydryl content in fish sausage due to less irradiation-induced oxidation [131]. Along with polysaccharides, UV could lead to better gel hardness, compactness and three-dimensional networks of surimi gel in comparison with control gel due to fewer increases in carbonyls and surface hydrophobicity. However, the use of UV for 20 min boosted gel intensity by 20% compared to the traditional gelation process [132]. It can be concluded that UV light may strengthen the crosslinking of existing myosin chains in the actomyosin of surimi structure [132]. However, due to the high UV light sensitivity of cysteine in the myosin, an excessive increase in treatment time would dramatically reduce the gelling performance [129]. Cardoso, Mendes, Vaz-Pires and Nunes [116] have investigated the impact of simultaneous addition of konjac flour (1%) and 40 min UV radiation at 250 nm, which increased the texture and surimi gel strength. The application of UV light with konjac flour produced maximum gel strength (63.2 N mm) and springiness (0.84), with the increased WHC. This study indicates that the use of UV alone may not have a substantial effect on the texture consistency of prepared protein gels.

### 5.5. Ohmic Heating

Ohmic heating is a technique in which alternating electric current passes through a food mixture. The food materials serve as resistors and their temperature is increased based on the Joule effect. The food materials serve as resistors and their temperatures vary due to the resulting air currents [133]. An increase in temperature can influence the micro- and macro-structure and lead to certain phenomena such as water movement, protein coagulation and starch gelatinization. Meanwhile, the use of ohmic heating also prevents the activation of polyphenoloxidase and lipoxygenase, which are responsible for oxidative changes. Surimi added with egg white and treated with ohmic heating significantly reduced the protolysis compared to the water bath heating system. In addition, ohmic heating also increased the stability of cysteine, which is a key part of myosin and responsible for oxidative changes [134]. Moreover, the application of ohmic heating to the surimi gel also significantly reduced the formation of disulfide bonds and resulted in an increase in total sulfhydryl content, which indicates the decline in the proteolysis process and results in proper MP interactions and gel formation [135].

It is also a constructive aim to take the non-thermal impacts of ohmic applications into account, particularly when an alternating current is applied to food materials [136]. While it is well known that ohmic heating effects on the textural and structural properties of food items have been observed, it depends on the characteristics of the food and the conditions of the process, including the process temperature, applied voltage and frequency [129,137].

Kulawik [138] investigated the textural and structural consistency of salt-added salmon gel using an ohmic treatment. Based on the findings by Tadpitchayangkoon, Park and Yongsawatdigul [135], ohmic heating at 45 °C for 5 min before salting slightly improved the product appearance and quality. Another study demonstrated that ohmic heating at 90 °C with 60 Hz and 9 V/cm resulted in increased maximal cutting strength of surimi gel (15.6 N) as compared to the traditional heating system (20.4 N). The study showed that the lower temperature processing of surimi gel resulted in less denaturation and oxidation of the MPs in the various fish species [139]. The textural characteristics of the thawed fish are found to be similar to those of fresh tilapia [140]. One of the reasons this technology is considered superior to conventional ones is because of its better influence on surimi textural properties. A benefit of ohmic heating is that it will make the process of heating spread equally across the sample [141]. Poor consistency or gel strength of fish can be obtained from an insufficient heating rate in traditional and slower heating methods [136]. Traditional processing with slow water bath cooking resulted in poor quality surimi gel, while ohmic heating of surimi improved the quality of the gel matrix, texture and strength significantly [139].

This research enlightened the value of ohmic application in improving the textural consistency of gel products. In addition, ohmic heating increased the textural attributes such as hardness, springiness and gumminess of Japanese whiting surimi gel at different temperatures (3, 60 and 160 °C/min), which developed a harder and more compact gel. It is obvious that the ohmic heating system provides better results relative to conventional water bath heating [142]. [Chai and Park [143]] reported that variations in time-temperature combinations and heating were the primary factors in varied textural properties during ohmic and water bath processed gel products. The applied voltage and product formulation were shown to be effective parameters to analyze the gel strength. Moreover, the role of modern processing technologies in enhanced textural properties of surimi gel is shown in Table 2. It can be concluded from the current literature that ohmic heating could be effectively used to enhance the textural and functional properties of fish and seafood gel products.

## 6. Conclusions

This study emphasizes the importance of advanced processing techniques (HPP, ultrasound, MW and UV light) for improved textural properties of surimi gel. Food hydrocolloid-based antioxidants in conjunction with innovative processing technologies had a major impact on the protein cross-linking rate, retarding oxidation and denaturation, which make the gels more compact and denser in terms of structure. It would be very possible in the enhanced textural, whitening, antioxidant and sensory characteristics of protein-based gels. The textural, gelling and structural properties of gels prepared by modern processing technologies are better than those produced by traditional thermal processes. The effect of antioxidants combined with modern processing techniques can improve the stability in protein by preventing the oxidative changes in myosin proteins, denaturation and aggregation as compared to the conventional gelation method. These modern processing techniques are also effective at improving the textural properties by enhancing the amino acids cross-linking, intermolecular interactions and hydrogen bindings than water bath gelation process. Besides all these benefits, there is a need to establish appropriate methods for how to optimize conditions before performing these modern techniques on surimi gel. This has the potential to reverse many of the negative changes that have occurred in surimi and other food products, as well as improve their safety. There has not been enough literature reported about the effects of novel processing methods, such as pulsed X-rays, pulsed illumination, pulsed electric field and atmospheric cold plasma on the consistency and efficiency of seafood protein gels. Furthermore, it is unclear whether a combination of different new technologies influences the cross-linking ability of protein molecules in heat-induced surimi gels.

Most research on non-thermal processing of food by various technologies has been done on the laboratory and pilot scale, where the process conditions, such as time, pressure, temperature and volume of food material might vary at the industrial or commercial scale. Thus, developing and maintaining optimal conditions for large-scale industrial activity is important. These techniques require massive initial expenditure, followed by large maintenance expenses. Therefore, these are the reasons that such innovations are not applied at the industrial scale. More studies are required on these approaches in combination with different food-based additives to analyze the protein cross-linking for a stable gel network and high-quality and stable hydrogels for the delivery of bioactive compounds. There should be logical and well-crafted hypotheses on emerging technology. Moreover, special attention is required to analyze and optimize the parameters of such advanced technologies for large-scale production.

## Figures and Tables

**Table 2:** Role of modern processing techniques on enhanced textural properties of surimi gel.

Processing Technique	Additives	Role	Results	Reference
HPP	Kappa-carrageenan (KC)	Oligosaccharids, antioxidants and cryoprotectants	Surimi gel treated with KC showed better WHC and gel strength on HPP (300 MPa), by improving the water state and structural properties.	[53]
HPP	mTGase	Microbial	The mTGase treated surimi gel showed increased fracture stress, strain and gel strength when cooked at 300 MPa processing.	[144]
Ultrasonication	Soybean polysaccharide (SSPS)	Polysaccharide, antioxidants, funtional and gelling	The SSPS added surimi gel revealed enhanced whiteness and gelling properties during frozen storage combined with ultrasonication.	[112]
Ultrasonication	Wheat gluten (WG)	Protien additive, functional and gelling	Wheat gluten-SPI gels with ultrasonication led to increase in textural properties by improving β-sheets, decreased α-helices and β-turns.	[13]
Microwave	NaCl	Functional and mechanical	The mechanical, structural and textural characteristics of NaCl-treated surimi gel improved after 80 s heating of MW (15 W/g).	[145]
Microwave	Konjac glucomannan (KGM)	Oligosaccharide, antioxidant and functional	Microwave heated KGM surimi gel displayed better starching of protein molecules and dense KGM-protein network.	[124]
Ultraviolet	Konjac flour (KF)	Dietary fiber, gelling	KF (1%) and 250 nm UV for 40 min increased the gel hardness (63.2 N) and springiness (0.84).	[116]
Ohmic heating	Corn starch (CS)	Carbohydrate, functional and thermo-stable	CS-surimi gel displayed inferior gel network due to starch gelatinization. But the control surimi gel exhibited improved hardness and gel strength when processed with ohmic technique as compared to water bath cooked gel.	[142]
Ohmic heating	Diced carrot (DC)	Sensory and functional	DC added surimi of Pacific whiting (PW) and Alaska Pollock (AP) reported increased hardness and cohesiveness when ohmically heated at 90 °C.	[24]
